# Application of Industrial NF and RO Membranes in Separation of Post-Fermentation Solutions: Preliminary Study

**DOI:** 10.3390/ma18122779

**Published:** 2025-06-12

**Authors:** Wirginia Tomczak, Marek Gryta, Sławomir Żak, Monika Daniluk

**Affiliations:** 1 Faculty of Chemical Technology and Engineering, Bydgoszcz University of Science and Technology, 3 Seminaryjna Street, 85-326 Bydgoszcz, Poland; zak@pbs.edu.pl (S.Ż.); monika.daniluk@pbs.edu.pl (M.D.); 2 Faculty of Chemical Technology and Engineering, West Pomeranian University of Technology in Szczecin, ul. Pułaskiego 10, 70-322 Szczecin, Poland

**Keywords:** biotechnology, downstream processing, fermentation broth, industrial membrane, nanofiltration, polymeric membrane, separation properties

## Abstract

The focus of this work was to perform a preliminary study on the suitability of commercially available nanofiltration (NF) and reverse osmosis (RO) membranes for the separation of 1,3-propanediol (1,3-PD) post-fermentation solutions. The experiments were conducted with the use of AFC30 and AFC99 (PCI Membrane System Inc., Milford, OH, USA) as well as BW30 membranes (Dow FilmTec Co., Midland, MI, USA) and various feed solutions: selected compounds of fermentation broths, and synthetic and real fermentation broths. Firstly, it was found that for pure water, the AFC30 membrane was characterized by the highest performance. It clearly indicated that the membrane is the most open membrane and is characterized by a more porous structure. In turn, the lowest flux was noted for the AFC99 membrane. Studies performed with the use of synthetic broth found that for the BW30 membrane, the order in which the rejection coefficient (R) was obtained was glycerol~lactic acid > 1,3-propanediol > acetic acid. It clearly confirmed that the R increased with the molecular weight (MW) of the solution compounds. With regard to ions, it was found that SO_4_^2−^ and PO_4_^3−^ is characterized by higher R than Cl^−^ and NO_3_^−^ ions. Multivalent ions are characterized by higher charge density, hydrated radius, hydration energy and MW. Finally, experiments performed with the use of the AFC30 membrane and real broths showed that the membrane ensured almost complete separation of 1,3-PD. With regard to organic acid, the separation performance was as follows: succinic acid > lactic acid > butyric acid > acetic acid > formic acid. It has been documented that the AFC30 membrane can be successfully used to concentrate the following ions: SO_4_^2−^, PO_4_^3−^, NO_3_^−^ and Na^+^. Hence, most of the medium used for the fermentation process was retained by the membrane and may be reused, which is crucial for the scaling up of the process and reducing the total technology cost. With regard to the obtained permeate, it can be subsequently purified by other methods, such as distillation or ion exchange. For further development of the tested process, determining the retention degree for 1,3-PD and other solutes during long-term separation of real broth is necessary.

## 1. Introduction

Nowadays, industrial biotechnology offers the production of more and more various valuable chemicals from a wide range of feedstocks. For instance, Dupont manufactured 1,3-propanediol (1,3-PD) via the fermentation process, which allowed up to 40% of the energy consumption to be saved compared to the production of this vital value-added material [1,2,3] based on the conventional fossil fuel-based method [4]. Undoubtedly, biological production should be characterized by a high selectivity. Furthermore, a high level of product purity is expected. For this purpose, downstream processing (DSP) that is aimed at recovering the target product from a bioreactor effluent can be applied. Presently, significant research focus is being placed on DSP.

It is very important to note that DSP should ensure minimum cost and environmental issues in relation to the amount of the desired product [5]. It is a particular challenge if DSP requires multistage unit operations. Unfortunately, as has been indicated by Xiu and Zeng [6], the downstream processing for fermentative 1,3-PD contributes up to 70% of the overall production costs. Moreover, it is essential to mention that up to now, the recovery of 1,3-PD from fermentation broths has not received much attention [7]. Hence, it still needs to be further investigated.

In the first DSP stage, separation of the microorganisms from the broth is required. Preliminary clarification can be carried out by various techniques, for instance microfiltration (MF) [8,9,10], ultrafiltration (UF) [10,11,12] and centrifugation [13]. After the above-mentioned operations, the solution contains, in addition to main product such as 1,3-PD, salts and carboxylic acids. The 1,3-PD present in a solution can be separated by several stages of distillation/rectification [14]. This method is limited by the fact that the broth is diluted, which is related to the high concentration costs, and that it contains salts that crystallize inside the distillation apparatus during concentration. For these reasons, for further processing, a pre-concentrated and desalted 1,3-PD solution should be obtained. Worthy of note is that, during fermentation, NaOH is added to the broth to maintain pH at 7, which increases the efficiency of fermentation; however, such operation also increases the salt content [15].

It is well known that pressure-driven membrane processes are increasingly applied in biotechnology for concentration and/or purification of biological solutions [12,16]. For instance, nanofiltration (NF) and reverse osmosis (RO) can be successfully used for the separation of pre-treated fermentation broths. NF membranes mostly possess negative charge at neutral condition [16,17,18] and their pores range from 0.5 to 10 nm [19]. Fundamentally, they can be applied for the separation of organics from salts as well as multivalent ions from univalent ones [20]. Many researchers have made remarkable achievements in the application of polymeric NF membranes for primary research due to their significant advantages, for instance their high performance and mechanical strength as well as longer durability [19,21,22]. Semi-aromatic piperazine-based polyamide NF270 and aromatic polyamide ES10 membranes at a solution pH of 7 retained organic acids in the range of 60–90% and over 90%, respectively [23]. The above-mentioned difference was explained by the different molecular weight cut-off (MWCO) values (100 Da and 200–300 Da for the ES10 and NF270 membranes, respectively). However, it is worth emphasizing that the feed composition also has an impact on the rejection degree. For instance, retention of salt and organic acids of over 90% was achieved in studies performed with the use of real fermentation broth and NF270 membranes [15]. It has been demonstrated that the membranes did not retain either 1,3-PD or lower molar mass components, such as formic and acetic acids, or the following ions: K^+^, Na^+^ and Cl^−^. Similar results were obtained in another study [24], wherein the mechanisms of separation of real broth components by the NF270 membranes were investigated.

In order to concentrate the permeate obtained in the NF process, the RO process can be applied. The RO membranes compared with NF membranes possess a less loose structure [25]. It should be pointed out that in the last five years, several review publications have been focused on the fundamentals of both the NF, e.g., [19,26,27], and RO processes, e.g., [28,29,30]. The RO process is the primary membrane-based technology for desalination and water purification. However, studies on the separation of organic solutions by RO are limited. The BW30 membranes have been used for the concentration of 2,3-butanediol and acetate [31] and removal of multiple pesticides from water [32].

AFC30, AFC99 and BW30 membranes are the most commonly investigated industrial membranes for nanofiltration and reverse osmosis. The basic properties and performance parameters of the above-mentioned membranes have been presented in the studies in [33,34,35,36,37,38,39]. These membranes play a significant role in various applications. For instance, the AFC30 membranes have been used for the treatment of effluents from a membrane bioreactor [40], the separation of antibiotics [41], the recovery of contaminated single-phase acidic detergents [42] and the removal of hardness in wastewater effluent [43] and transition metals present in effluents from in a totally chlorine-free bleaching plant [44]. In turn, the AFC99 membranes have been considerably applied to the treatment of seawater [45] and removal of amines from wastewater [46]. In general, the broth turbidity is high, which, despite preliminary clarification, may be a great challenge when using flat membranes (such as NF270 or BW30) mounted in spirally wound modules. To separate turbid solutions, tubular membranes can be successfully applied. In the discussed case, AFC30 membranes can be used for the NF process and AFC99 membranes for the RO process.

Recently, attempts have been made to explore the potential of NF and RO processes in the separation of 1,3-PD from fermentation broths, e.g., [15,24,47,48]. The rejection of broth components, especially organic solutes, is significantly influenced not only by the type of process applied (NF or RO), but also by the type of membrane-forming polymers. It has been found that RO cellulose acetate membranes do not retain 1,3-PD and ensure the high rejection of salts and organic acids [49]. Obviously, organic solutes can also be adsorbed by the polymeric matrix, which causes changes in the membrane’s performance over time [15,24]. Such changes are particularly well observed when using large membrane modules; hence, in the current study, the pilot installation was used. Finally, it should be pointed out that even small changes in the composition of the membrane’s active layer can affect the effectiveness of the separation process. The aim of this work was to determine the suitability of commercially available NF (AFC30) and RO (AFC99 and BW30) membranes for the separation of 1,3-PD post-fermentation solutions. The findings may have a significant impact on the development of the downstream processing of 1,3-PD solutions.

## 2. Materials and Methods

### 2.1. Feed Solutions

Within this study, experiments were carried out using various feed solutions: selected compounds of fermentation broths, and synthetic and real fermentation broths.

In the first test of membrane properties, the following standard charged and uncharged solutions were used as a feed: sodium chloride (NaCl) 2 g/L, citric acid (C_6_H_8_O_7_) 1 g/L, glycerol (C_3_H_8_O_3_) 1 g/L and 1,3 propanediol (C_3_H_8_O_2_) 1 g/L.

Subsequently, the separation of the synthetic fermentation broth was thoroughly investigated. The prepared broth consisted of the following organic compounds: glycerol 1.1 g/L, 1,3-propanodiol 1 g/L, lactic acid (C_3_H_6_O_3_) 0.31 g/L and acetic acid (C_2_H_4_O_2_) 0.5 g/L; it also contained the following ions: Cl^−^ 0.68 g/L, NO_3_^−^ 0.001 g/L, PO_4_^3−^ 0.33 g/L, SO_4_^2−^ 0.81 g/L, Na^+^ 0.005 g/L, NH_4_^+^ 0.52 g/L, K^+^ 0.16 g/L, Ca^2+^ 0.004 g/L and Mg^2+^ 0.21 g/L.

Finally, the experiments were performed with the use of real 1,3-PD fermentation broths. The fermentation process was conducted in a LiFlusGX bioreactor (Biotron Inc., Bucheon, Republic of Korea). The studies were performed with *Citrobacter freundii* bacteria (Poznań University of Life Science, Poland). Fresh bacteria cultures were inoculated under sterile conditions in the bioreactor (bacteria culture comprised 5% of the total reactor volume). The medium (2 L) contained glycerol (40 g/L), 3.4 g of K_2_HPO_4_, 0.7 g of KH_2_PO_4_, 1.1 g of (NH_4_)_2_SO_4_, 0.58 g of MgSO_4_∙7H_2_O, 0.013 g of CoCl_2_∙6H_2_O, 2.0 g of yeast extract, 2.5 g of peptone K and 1.5 g of meat extract in 1 L of distilled water. About 40–44 h fermentation processes were performed with agitation at 150 ± 5 min^−1^ and the incubation temperature equal to 303 K. The pH value was constant and equal to 7.0 (NaOH dosing).

The fermentation process was repeated many times. The obtained broth was purified by sedimentation for 10 h and frozen for storage. Finally, for NF studies, all post-fermentation solutions were mixed and 35 L of solution was obtained. Chemical species in fermentation broths and their associated properties are described in the study in [24].

### 2.2. Experimental Setup

**Figure 1:**
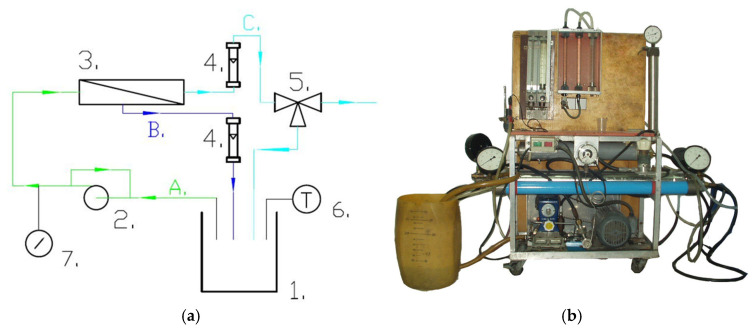
NF/RO pilot plant: (**a**) Scheme: 1—feed tank; 2—piston pump with by-pass; 3—tubular membrane module; 4—rotameters; 5—three-way cock; 6—electronic thermometer; 7—manometer; A—feed; B—retentate; C—permeate. (**b**) Photo of installation.

The NF process was carried out on the pilot plant presented in Figure 1. It was performed at a constant temperature of 303 K, a feed flow equal to 640 +/− 20 L/h and a transmembrane pressure (TMP) in the range between 5 and 30 bar.

The permeate flux [LMH] was obtained based on the following equation:

(1) $$J=\frac{V}{S\cdot t}$$

where V [L]—cumulative permeate volume; S [m^2^]—effective membrane surface area; t [h]—filtration duration.

The rejection efficiency R [%] was assessed based on the following equation:

(2) $$R=1-\frac{C_{p}}{C_{F}}\cdot 100\%$$

where C_P_ [mg/L] and C_F_ [mg/L]—concentrations of the permeate and feed, respectively.

### 2.3. Industrial Membranes

As indicated in the Introduction Section, polymeric membranes provide several significant advantages. Hence, in the current studies, three types of industrial polymeric membranes were used: AFC30 and AFC99 (PCI Membrane System Inc., Milford, OH, USA) as well as BW30 (Dow FilmTec Co., Midland, MI, USA).

A summary of the membrane characteristics is shown in Table 1. The membranes used in this study consist of an aromatic polyamide skin layer.

### 2.4. Analytical Methods

The rejection degree was calculated based on the components’ concentration in the feed and permeate. Liquid chromatography (HPLC) (organic compounds) and ion chromatography (inorganic compounds) were used for the analysis. The analytical methods were thoroughly described in previous studies [24,49,50,51].

## 3. Results and Discussion

### 3.1. Separation of Fermentation Broth Compounds

**Figure 2:**
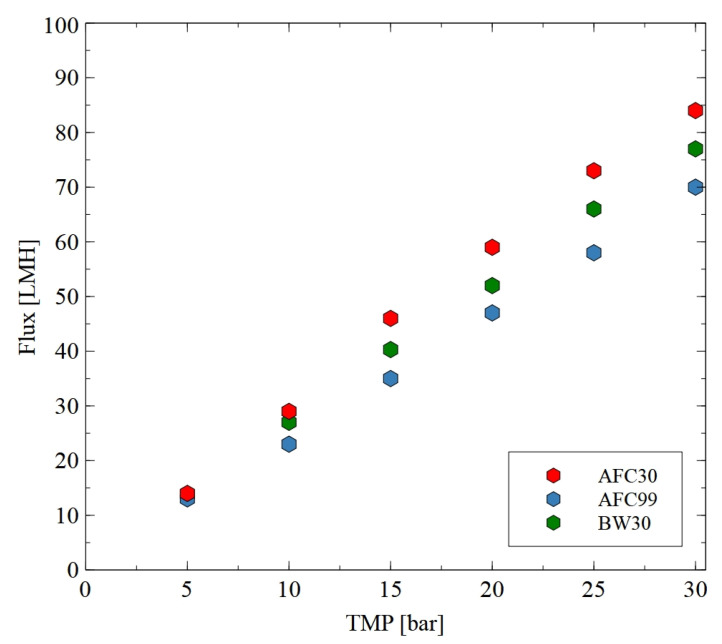
Impact of transmembrane pressure on the permeate flux for the AFC30, ACF99 and BW30 membranes.

**Figure 3:**
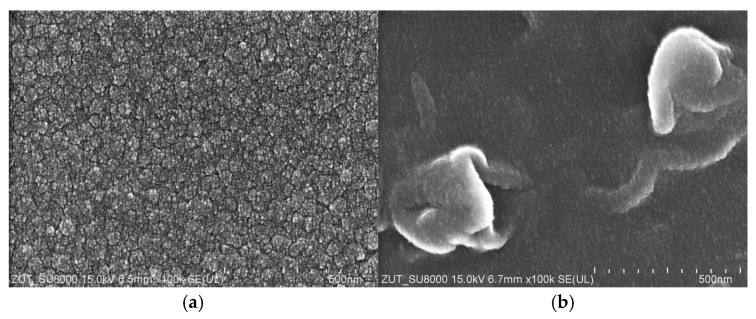
SEM images of membrane surface: (**a**) AFC30 nanofiltration; (**b**) BW30 reverse osmosis. Magnification ×100k.

Hydrophilic thin-film composite membranes made from polyamide were used in the current study. These membranes are characterized by some differences in their structure, which have a significant impact on their performance [37]. To determine the water permeability of the membranes, the studies were performed using distilled water as a feed (Figure 2). The applied TMP was in the range from 5 to 30 bar. Obviously, the permeate flux increased linearly with the TMP for all membranes used. As indicated in [52], higher pressure led to more solvent being pushed towards the membrane surface and, consequently, at higher pressure, higher values of flux were noted. For instance, at the TMP of 30 bar, the flux was equal to 70, 77 and 84 LMH for the AFC30, BW30 and AFC99 membranes, respectively. It is important to note that the obtained results are consistent with the values provided by the membrane manufacturers [33,34,35,36]. The SEM observations confirmed that the AFC30 membrane is the most open membrane and is characterized by a more porous structure (Figure 3a). In contrast, the reverse osmosis membranes have a more compact active layer (Figure 3b), which increases flow resistance and consequently reduces permeate flux. Worthy of note is that similar values of water flux for the AFC30 membrane at the same TMP range were obtained in a previous study [40], wherein the treatment of effluents from a membrane bioreactor was investigated. Moreover, a slightly high performance of the AFC membrane was noted in a study [53] focused on the NF of highly concentrated salt solutions up to seawater salinity.

**Figure 4:**
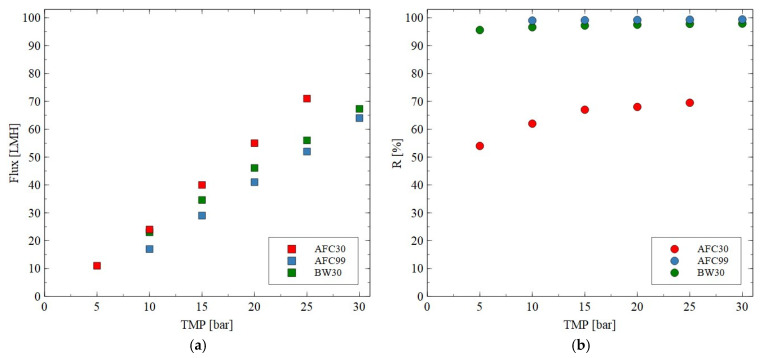
Impact of transmembrane pressure on the (**a**) permeate flux and (**b**) rejection efficiency for the AFC30, AFC99 and BW30 membranes. Feed: NaCl solution (2 g/L).

It was found that for each membrane type the presence of monovalent NaCl in the feed solution led to a decrease in the membrane performance (Figure 4). For instance, for the AFC30 membrane at TMP of 20 bar, the flux for pure water and NaCl solution was equal to 59 LMH and 55 LMH, respectively. Obviously, the noted reduction can be explained by the osmotic effect caused by the salt concentration, which affected the driving force for the flow through the membranes used. Such a phenomenon for the NF process has been obtained and discussed in several previously published papers, e.g., [54,55]. Importantly, based on the results, it can be seen that the increase in TMP led to an increase in the performance of all membranes tested. The noted NaCl rejection for the AFC30 membrane was equal to 60%, which is in line with the specification provided by the manufacturer [33]. The highest NaCl rejection efficiency was obtained for the AFC99 and BW30 membranes. Indeed, it was equal to almost 100%, which is promising for their application in both the desalination process and the separation of fermentation broths. The order in which R was noted is AFC99 > BW30 > AFC30. It is important to note that the NaCl retention for the AFC30 membranes increased strongly by increasing the TMP due to the increase in solvent flux. This finding is in good agreement with the experimental results presented in [23,53,56].

**Figure 5:**
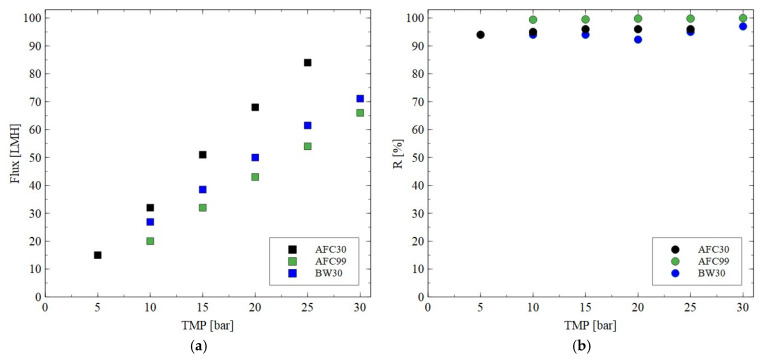
Impact of transmembrane pressure on the (**a**) permeate flux and (**b**) rejection efficiency for the AFC30, AFC99 and BW30 membranes. Feed: citric acid solution (1 g/L).

It should be noted that during the fermentation process, in addition to the target product, organic acids containing various impurities like a substrate or mineral salts are generated [57]. One of the most important organic acids produced during the glycerol fermentation is citric acid. The impact of the TMP on the permeate flux and retention degree for this by-product is shown in Figure 5. As in the case of the NF of NaCl solutions, it has been shown that the increase in the TMP allowed the permeate flux to be increased. However, the highest process performance was obtained for the AFC30 membrane, which confirmed the previously presented finding that this membrane is characterized by a more porous structure compared to other ones. In addition, it has been found that all membranes tested in the current study ensured a retention degree higher than 95%. Worthy of note is that a similar retention degree for citric acid was obtained in the study in [58], wherein the nanofiltration NF270 membrane ensured R equal to almost 100%.

Based on the above presented and discussed experimental results, the next stages of the research were carried out mainly using the AFC30 and BW30 membranes.

**Figure 6:**
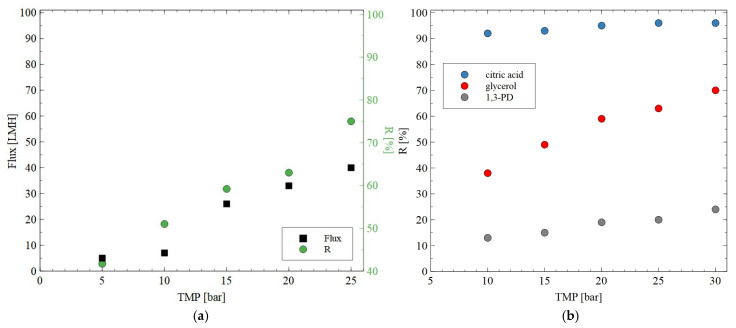
Impact of transmembrane pressure on the (**a**) permeate flux and rejection efficiency for glycerol (feed: 0.5% glycerol + 0.5% citric acid + 0.2% MgSO_4_) and (**b**) rejection of 1,3-PD, glycerol and citric acid (feed: 0.5% glycerol + 0.5% citric acid + 2% 1,3-PD). The AFC30 membrane.

In the next step of the presented research, the impact of the TMP on the permeate flux and rejection coefficient for glycerol during the NF of the solution containing glycerol (0.5%) + citric acid (0.5%) + MgSO_4_ (0.2%) was studied. For this purpose, the AFC30 membrane was used. As demonstrated in Figure 6a, increasing TMP led to an increase in both the permeate flux and the rejection degree for glycerol. It is interesting to note that at TMP of 25 bar, the R was equal to 75%. However, most of the 1,3-PD passed through the membranes (Figure 6b). The salts and glycerol can be returned to the bioreactor and used as nutrients [46].

### 3.2. Separation of Synthetic Fermentation Broth

**Figure 7:**
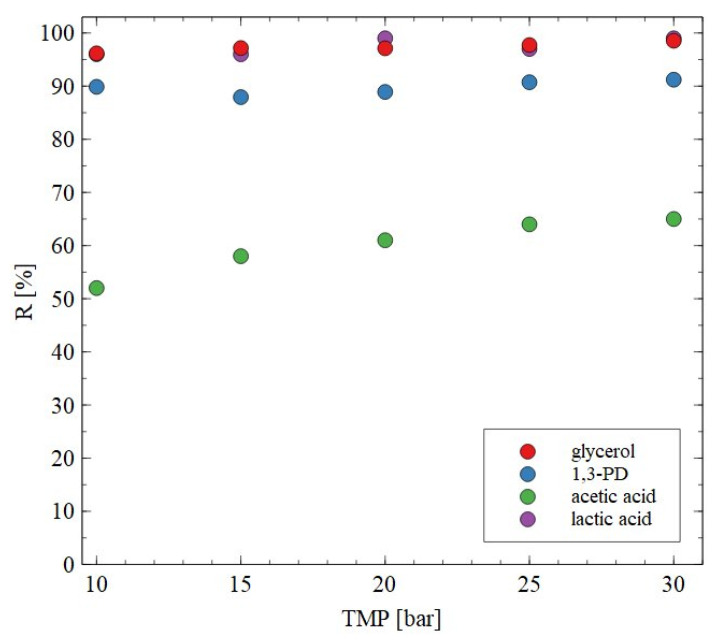
Impact of transmembrane pressure on the rejection efficiency. Feed: synthetic fermentation broth. BW30 membrane.

**Figure 8:**
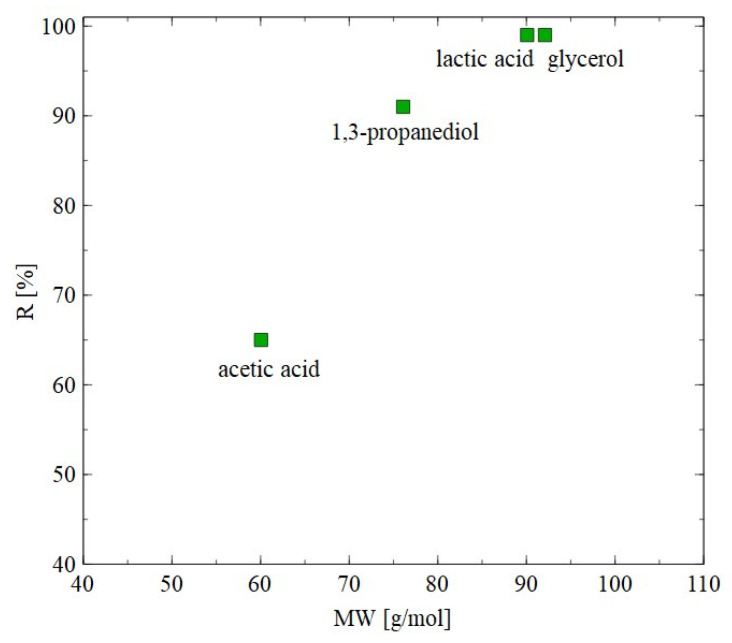
Impact of molecular weight on the rejection efficiency. BW30 membrane. TMP = 30 bar.

The AFC30 membranes retained most of the salts and organic acids while 1,3-PD was present in the permeate (Figure 4, Figure 5 and Figure 6). Since the obtained permeate is diluted, its concentration with the use of RO membranes is beneficial. In order to investigate the separation of synthetic 1,3-PD separation broth, the BW30 membrane was used. Figure 7 shows the retention degree for organic compounds present in the feed. First of all, it was found that regardless of the applied TMP, almost all lactic acid and glycerol was rejected by the membrane. Indeed, the noted R was in the range from 96 to 99%. A slightly lower R (about 90%) was recorded for 1,3-PD. Finally, the lowest R was observed for acetic acid; however, it slightly increased with TMP. Indeed, it was from 52 to 65% for TMP of 10 to 30 bar, respectively. Finally, it should be indicated that for the BW30 membrane, the order in which R was obtained was glycerol~lactic acid > 1,3-propanediol > acetic acid. This can be explained by the fact that the rejection efficiency increased with the molecular weight (Figure 8). This indicates that the separation mechanism with the use of the BW30 membrane was based mainly on the sieving effect. Hence, it can be indicated that the RO process makes it possible to concentrate the 1,3-PD solution, characterized by the presence of other organic components of fermentation broth.

**Figure 9:**
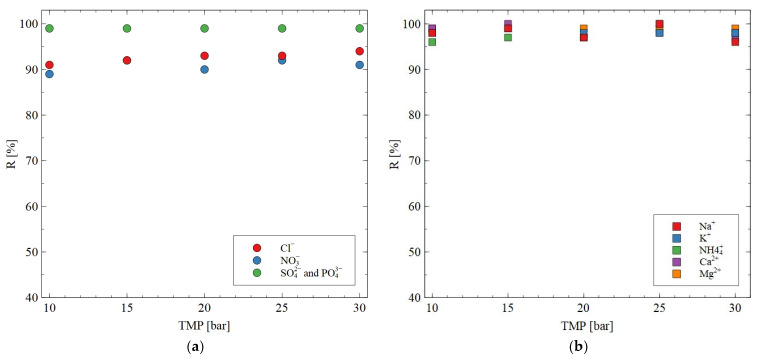
Impact of transmembrane pressure on the rejection efficiency for (**a**) anions; (**b**) cations. Feed: synthetic fermentation broth. The BW30 membrane.

The AFC30 membranes did not retain all the salts present in the fermentation broth; hence, some of them were concentrated with the use of the RO membranes. The rejection efficiency as a function of TMP for ions present in the synthetic fermentation broth is shown in Figure 9. It was found that SO_4_^2−^ and PO_4_^3−^ are characterized by higher R than Cl^−^ and NO_3_^−^ ions. This can be explained by the fact that multivalent ions are characterized by a higher (i) charge density, (ii) hydrated radius, (iii) hydration energy and (iv) molecular weight (Table 2). This result indicated the significant role of both the sieving effect and Donnan exclusion during ion separation. With regard to cations, it important to note that for all applied TMPs, the R was higher than 95%. Hence, it can be concluded that the BW30 membrane has been proven to be successful in almost complete cation retention. Based on this finding, it can be assumed that since the pH solution was neutral, the membrane was negatively charged.

**Figure 10:**
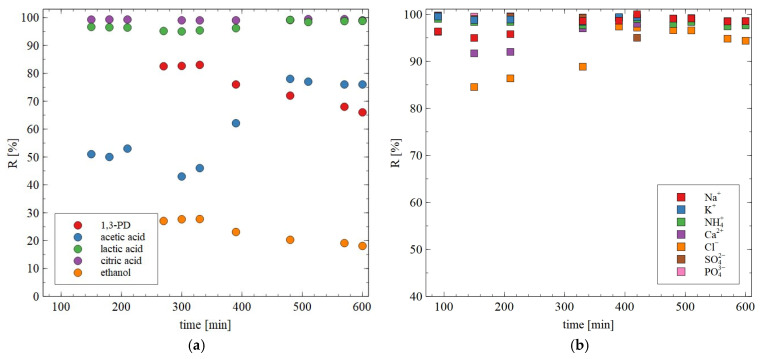
Changes in the retention degree during the RO process for (**a**) 1,3-PD, ethanol and acids; (**b**) ions. Feed (g/L): K_2_HPO_4_ (3.4), KH_2_PO_4_ (1.3), (NH_4_)_2_SO_4_ (2.0), MgSO_4_·7H_2_O (0.4), CaCl_2_ (0.076), citric acid (2.34), lactic acid (1.3), acetic acid (1.96), 1,3-PD (8.5) and ethanol (0.65). The BW30 membrane.

During long-term exploitation of membranes, their properties may change. The noted values of rejection degree during long-term studies are shown in Figure 10. Although the retention degree of most components of the broths changed slightly, the retention of 1,3-PD during 10 h of the RO process run decreased from 83 to 66%. This finding indicates that BW30 membranes allow significantly purified 1,3-PD solution to be obtained, which also contains ethanol and acetic acid. Worthy of note is that these more volatile components can be separated during distillation.

### 3.3. Separation of Real Fermentation Broth

With regard to multicomponent solutions, the separation efficiency depends not only on the membrane properties but also on the interactions between the solutes. For this reason, in the last stage of the current study, the real fermentation broth was separated by the AFC30 membranes, and then the obtained permeate was separated using the BW30 membranes.

**Figure 11:**
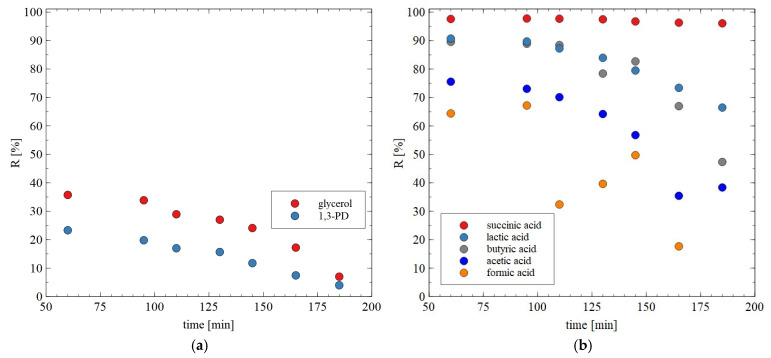
The effect of process time on the rejection efficiency for (**a**) glycerol and 1,3-PD; (**b**) organic acids during the NF of real fermentation broths. The AFC30 membrane.

**Figure 12:**
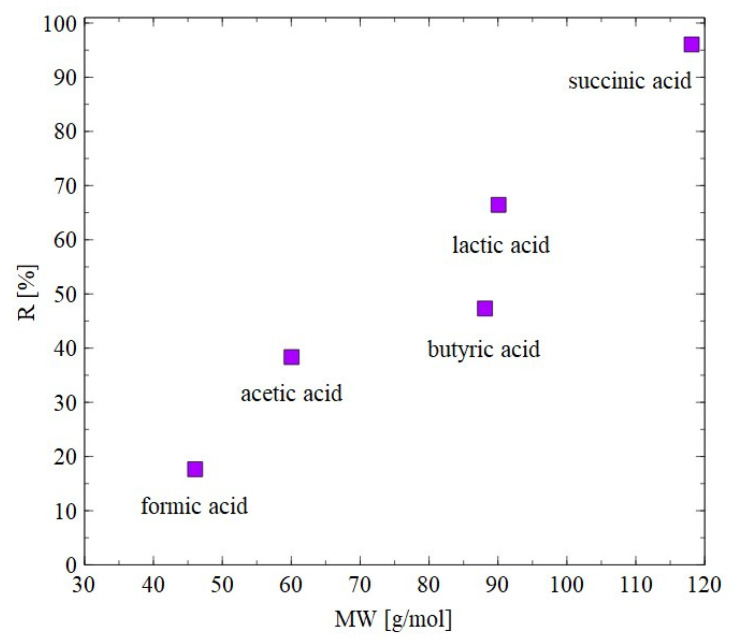
Impact of molecular weight on the rejection efficiency. The AFC30 membrane.

The results of separation of 1,3-PD fermentation broth with the application of the AFC30 membrane are shown in Figure 11. It has been demonstrated that during the first 60 min of the process run, the rejection degrees for glycerol and 1,3-PD were similar to those obtained for the model broth (Figure 6b). However, retention for 1,3-PD and glycerol decreased after 3 h from 20 to 5% and from 40 to 8%, respectively (Figure 11a). With respect to organic acid, a much higher rejection was noted, ranging from 20 to 97% (Figure 11b). It has been noted that the order in which separation performance was obtained as follows: succinic acid > lactic acid > butyric acid > acetic acid > formic acid. It can be assumed that since the feed pH (equal to 7) was higher than the acid pKa (Table 3), they dissociated into ionic forms, which significantly improved the separation efficiency of NF membranes. Nevertheless, it has been determined that the coefficient R is strongly dependent on the molecular weight (MW) (Figure 12). For instance, for succinic acid the coefficient R was constant during the NF process and equal to 97%. On the contrary, it was observed that the retention of formic acid in the end of the experiment was lower than 20%. Therefore, it can be concluded that organic acids present in the real fermentation broth were mainly retained by a sieving effect. Worthy of note is that a very similar rejection sequence was obtained by Kand and Chang [61], wherein the removal of organic acid salts from simulated fermentation broth was studied. More precisely, the above-mentioned authors demonstrated that the use of the NF45 membrane ensured the R in the following order: sodium succinate > sodium lactate > sodium acetate > sodium formate.

**Figure 13:**
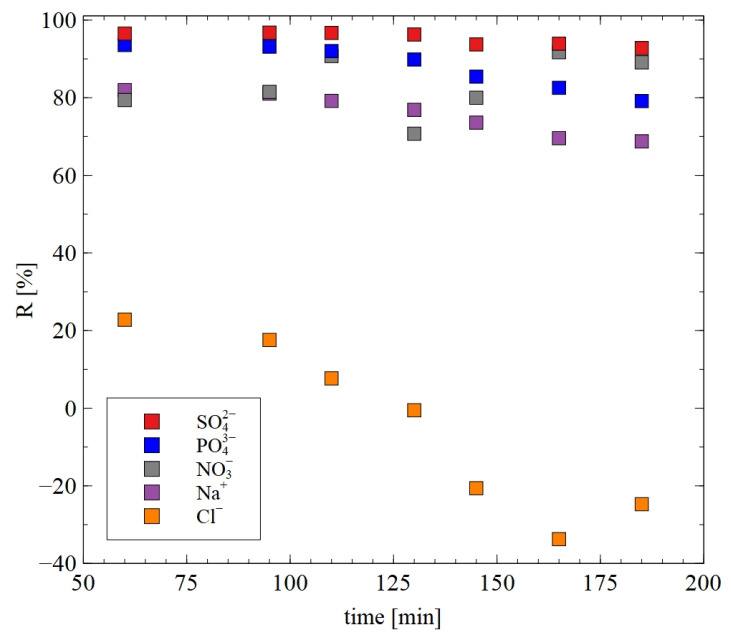
The rejection efficiency for ions during the NF of real fermentation broths. The AFC30 membrane.

To a much greater extent, the AFC30 membrane can reject the ions of salts presented in the real 1,3-PD fermentation broths. The results presented in Figure 13 substantiated the notion that with regard to anions, the R order was as follows: SO_4_^2−^ > PO_4_^3−^ > NO^3−^ > Cl^−^. As has been indicated above, it can be explained by the fact that SO_4_^2−^ and PO_4_^3−^ are characterized by higher charge density, hydrated radius, hydration energy and molecular weight. An important point which should be noted is that determining these effects is crucial to the scaling up of the process [18] and reducing the total technology cost.

**Figure 14:**
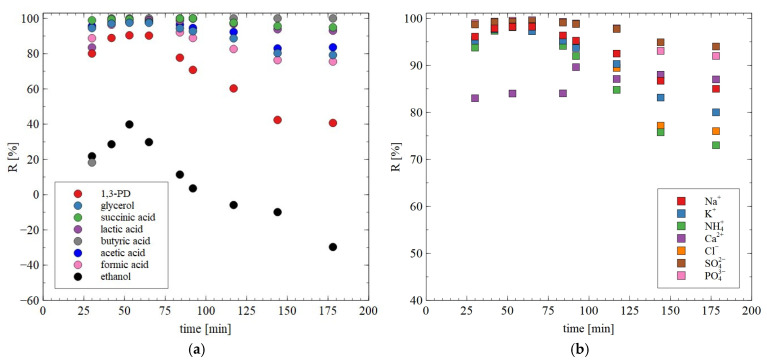
Changes in the retention degree during the RO process for (**a**) organic solute and (**b**) inorganic ions during the RO of NF permeate obtained from real fermentation broths. Feed: permeate obtained during the NF of real fermentation broths. The BW30 membrane.

The obtained NF permeate was subjected to further separation using the BW30 membranes. The noted rejection degree for most solutes was in the range of 80–100% (Figure 14). On the other hand, a significant decrease in R was noted for 1,3-PD. Indeed, after 2 h of the process run it decreased to 40%. This result confirms the conclusion that the BW30 membranes allow permeate containing mainly 1,3-PD to be obtained. This finding significantly facilitates further purification methods, such as distillation or ion exchange.

## 4. Conclusions

It is well known that nowadays, industrial biotechnology offers the production of various target chemicals from a wide range of feedstocks. However, in order to obtain a high level of product purity, downstream processing is required. It can be performed by the use of pressure-driven membrane processes, such as nanofiltration and reverse osmosis. The conducted studies have shown that commercially available NF (AFC30) and RO (BW30) membranes in two-stage separation (NF-RO) allow, from fermentation broth, a solution containing mainly 1,3-PD to be obtained.

During the NF process of synthetic broths, the order in which the rejection coefficient was obtained was glycerol~lactic acid > 1,3-propanediol > acetic acid. This demonstrated that the separation efficiency increased with the molecular weight of the solution compounds. With regard to ions, it was found that SO_4_^2−^ and PO_4_^3−^ are characterized by higher R than Cl^−^ and NO_3_^−^ ions. This can be explained by the fact that multivalent ions are characterized by a higher charge density, hydrated radius, hydration energy and MW. A similar impact of MW on the retention degree was found for the RO process with the use of the BW30 membrane.

Finally, experiments performed with the use of the AFC30 membrane and real broths made it possible to show that the membrane ensured almost complete separation of 1,3-PD. The BW30 membranes retained most of the solutes present in the broth. This allows for the concentration of the obtained NF permeate. However, during the RO process run, a reduction in the 1,3-PD retention was observed. Indeed, after 2 h of the process run, it decreased to 40%.

Finally, it can be concluded that the application of the two-stage membrane separation allows purified permeate containing mainly 1,3-PD to be obtained, which can be subjected to further purification methods, such as distillation or ion exchange.

Undoubtedly, as observed in the present study, changes in the membranes’ properties, progressing with the processes run, require further studies. For further development of the tested process, determining the retention degree for 1,3-PD and other solutes during long-term separation of real broth is necessary.

## Figures and Tables

**Table 1:** Membrane characteristics [33,34,35,36,37,38,39].

Parameter	Unit	AFC30	AFC99	BW30
Classification	-	NF	RO	RO
Module length	mm	1200	1200	1200
Module diameter	mm	100	100	100
Tubular membrane diameter	mm	12.3	12.3	-
Membrane surface	m^2^	0.99	0.99	2.6
Membrane-forming polymer	-	aromatic polyamide	aromatic polyamide	aromatic polyamide
Maximum process pressure	bar	60	64	41
Maximum process temperature	°C	60	80	45
Maximum feed flow	m^3^/h	1.5	1.5	1.4
Free chlorine resistance	mg/dm^3^	<0.1	<0.1	<0.1
pH range	-	1.5–9.5	1.5–12.0	2.0–11.0
Degree of desalination NaCl	%	70	99	99.5
Water contact angle	deg	31.0	49.5	58.0 ± 1.26
Hydrophilicity	-	4	4	4

**Table 2:** Properties of the anions present in the synthetic fermentation broth [59,60].

Anion	Hydrated Radius [nm]	Hydration Energy [kJ/mol]	Molecular Weight [g/mol]
Cl^−^	0.332	−340	35.45
NO_3_^−^	0.335	−300	62.00
SO_4_^2−^	0.379	−1080	96.06
PO_4_^3−^	0.339	−2765	94.97

**Table 3:** Properties of the organic acids present in synthetic fermentation broth.

Organic Acid	pKa	MW [g/mol]	Form in Fermentation Broth
succinic acid	4.21 and 5.64	118.09	negative
lactic acid	3.08	90.08	negative
butyric acid	4.82	88.11	negative
acetic acid	4.76	60.05	negative
formic acid	3.84	46.03	negative

## Data Availability

The original contributions presented in the study are included in the article; further inquiries can be directed to the corresponding authors.
